# Endosphere microbiome comparison between symptomatic and asymptomatic roots of *Brassica napus* infected with *Plasmodiophora brassicae*

**DOI:** 10.1371/journal.pone.0185907

**Published:** 2017-10-24

**Authors:** Ying Zhao, Zhixiao Gao, Binnian Tian, Kai Bi, Tao Chen, Huiquan Liu, Jiatao Xie, Jiasen Cheng, Yanping Fu, Daohong Jiang

**Affiliations:** 1 State Key Laboratory of Agricultural Microbiology, Huazhong Agricultural University, Wuhan, Hubei Province, P-R China; 2 The Provincial Key Lab of Plant Pathology of Hubei Province, College of Plant Science and Technology, Huazhong Agricultural University, Wuhan, Hubei Province, P-R China; 3 State Key Laboratory of Crop Stress Biology for Arid Areas, College of Plant Protection, Northwest Agriculture and Forestry University, Yangling, Shanxi, P-R China; Dong-A University, REPUBLIC OF KOREA

## Abstract

Clubroot caused by *Plasmodiophora brassicae*, is a severe disease of cruciferous crops that causes large hypertrophic galls in the roots. The plant microbiome is important for growth promotion and disease suppression. In this study, using 16S rRNA and internal transcribed spacer (ITS) sequencing techniques, we compared the endosphere microbiome of symptomatic and asymptomatic *B*. *napus* roots infected with *P*. *brassicae* collected from the same natural clubroot field. The results showed that the microbial population and its relative abundance in the asymptomatic roots was far higher than that in the symptomatic roots, and that many microorganisms in asymptomatic roots have biological control and plant growth promotion functions that may be related to clubroot symptoms. These results suggest the importance of the endosphere microbiome in clubroot disease and provide potential bio-control resources for its prevention.

## Introduction

Although studies have been conducted for many years, the plant microbiome has gained public attention as a new concept only recently [1, 2]. Turner et al. considered the plant microbiome to mainly include phyllosphere microorganisms, rhizosphere microorganisms, and endogenous microorganisms [3]. Edwards et al. clarified that spatial resolution distinguished three root-associated compartments: the endosphere (root interior), the rhizoplane (root surface), and the rhizosphere (soil close to the root surface). Compared to the rhizosphere and the rhizoplane, the endosphere is highly specific in terms of the microbial communities that it contains [4].

The plant microbiome has been identified as an important determinant of plant health [5]. In its capacity to do harm, the microbiome contains many plant pathogens such as *Magnaporthe oryzae*, *Sclerotinia scleroterum*, *Botrytis cinerea*, and *Fusarium* sp. In terms of benefits, among others, it contributes to the promotion of growth and resistance to disease [2, 5, 6]. Textbook examples of the microbiome contributing to plant growth promotion include the assistance by mycorrhiza and rhizobia to plants in phosphorus and nitrogen uptake [7, 8]. Some nitrogen-fixing bacteria belong to *Azorhizobium*, *Bradyrhizobium*, *Ensifer*, *Mesorhizobium*, *Rhizobium*, and *Sinorhizobium*n [9], and most mycorrhizae belong to the phylum Glomeromycota, which only comprises endophytes known as arbuscular mycorrhizal fungi [10, 11]. Many microorganisms in the microbiome have been used to prevent and control plant diseases. For example, *Trichoderma* controls a broad range of diseases such as *Fusarium* wilt, grey mold, and white rot disease [12, 13]; *Pseudomonas* can be used to control cotton blight and other soil-borne diseases [14, 15]; and *Streptomyces* has been useful in controlling *Phytophthora* root rot [16]. Overall, with increasing research on this topic, the plant microbiome is gaining more attention.

Clubroot is caused by a protist, *Plasmodiophora brassicae*, and is a significant disease in cruciferous crops [17, 18]. It endangers plants by forming finger, bar, or spherical galls in their roots. In a field survey conducted, in the same field contaminated with *P*. *brassicae*, we found that some rapeseed seedlings were seriously diseased with typical symptoms whereas some of the neighboring seedlings showed no obvious symptoms. One of the reasonable explanations for this phenomenon is the uneven distribution of the pathogen throughout the field; however, *P*. *brassicae* could be detected in the root cortical cells of asymptomatic plants using polymerase chain reaction (PCR) amplification. Therefore, symptoms manifestation may be associated with other factors. In previous studies, a higher frequency of *Curtobacterium flaccumfaciens* was observed in asymptomatic plants than that in symptomatic plants, which may contribute toward resistance to citrus variegated chlorosis (CVC) [19]. Asymptomatic or symptomatic CVC in citrus plants may have a relationship with the microorganism population balance (mainly *Methylobacterium* spp., *C*. *flaccumfaciens*, and *X*. *fastidiosa*) [20]. The frequency of bacterial isolates possessing various plant-beneficial properties was higher in asymptomatic citrus plants affected by Huanglongbing than that in symptomatic citrus plants [21]. Bruez indicated that diverse fungi and bacteria colonized the woody tissues of asymptomatic and symptomatic esca-diseased grapevines [22]. Therefore, we assume that the microbiome might be associated with the asymptomatic and symptomatic presentation of clubroot under certain conditions.

While 16S rRNA is the most suitable appraisal indicator for the systemic development and classification of bacteria, as the use of the internal transcribed spacer (ITS) region in fungal identification has also gradually increased [23, 24]. Sequencing of 16S rRNA and ITS has become an important means for studying the plant microbiome [25–29]. In this study, the root endosphere microbiome in symptomatic and asymptomatic seedlings was analyzed by sequencing the 16S rRNA and ITS sequencing to clarify the relationship between symptoms and microbiome.

## Materials and methods

### Preparation of samples for sequencing

*P*. *brassicae*-infected *B*. *napus* samples were collected (December 18, 2014) from a natural field large approximately 3 Mu (1 Mu = 0.0667 hectares) in Dangyang County, Hubei Province, PR China (30.50 N, 111.47 E), where clubroot disease had been occurring for five years. The plants were at the five- to ten-leaf stage. Two sample groups were collected: RS1 (i.e., asymptomatic root samples, no obvious swelling) and RS2 (i.e., symptomatic root samples, swollen). In each group, 30 samples were randomly collected and divided equally into three sub-groups. Each sub-group (including 10 plant roots) was regarded as a single sample for sequencing (in total six samples, denoted as RS1.1, RS1.2, RS1.3, RS2.1, RS2.2, and RS2.3).

### PCR and quantitative (q)-PCR detection

All roots were carefully washed with tap water before being peeled with a sterilized razor. The genomic DNA of all samples was extracted using the CTAB (hexadecyl trimethyl ammonium bromide) method [30]. DNA concentration and purity were monitored on 1% agarose gel. DNA was diluted to 1 ng/μL using sterile distilled water. The PCR protocol described by Wallenhammar and Arwidsson (2001) was used for detection of *P*. *brassicae* in RS1 and RS2 samples. PCR amplification was performed using the primers PbITS6: 5′-CAACGAGTCAGCTTGAATGC-3′ and PbITS7: 5′-TGTTTCGGCTAGGATGGTTC-3′ [31]. The conditions for PCR amplification included a denaturation step at 95°C for 5 min and 32 cycles of 94°C for 30 s, 59°C for 30 s, and 72°C for 1 min. The products of PCR amplification were sequence analyzed by BGI (Beijing Genomics Institute, Beijing).

For quantitative detection of *P*. *brassicae* in RS1 and RS2 samples, q-PCR analysis was performed using the primers Pb actin-F: 5′-CACCGACTACCTGATGAA-3′ and Pb actin-R: 5′-CAGCTTCTCCTTGATGTC-3′, according to the method described by Chen et al. [21]. The DNA samples were diluted to 500 ng/μL with sterile distilled water and 10-μL reactions were analyzed in triplicate using the CFX96 Real-Time PCR Detection System (Bio-Rad, USA). Each reaction mixture contained 5 μL of 2× SYBR Green Super mix (Bio-Rad, USA), 1 μL of sample DNA, 0.15 μL of forward primer, and 0.15 μL of reverse primer (10 μmol/L). Sterile distilled water was added to make up the final volume. The program was as follows: denaturation at 95°C for 3 min followed by 49 amplification cycles of 95°C for 15 s, 58.5°C for 20 s, and 72°C for 15 s. A melt curve was generated to verify the specificity of amplification from 65°C to 95°C with an increment of 0.5°C per cycle, with each cycle held for 5 s. The primer sequences are provided in S1 Table. The *B*. *napus* actin gene (primers: Bn actin-F: 5′-TGAAGATCAAGGTGGTCGCA-3′ and Bn actin-R: 5′-GAAGGCAGAAACACTTAGAAG-3′) was used as the internal control for normalization.

### 16S rRNA and ITS sequencing

Using the genomic DNA as a template, the 16S rRNA gene and ITS fragments were amplified using specific primers (515F: 5′-GTGCCAGCMGCCGCGGTAA-3′ and 806R: 5′-GGACTACHVGGGTWTCTAAT-3′ for 16S rRNA; ITS1-1F-F: 5′-CTTGGTCATTTAGAGGAAGTAA-3′ and ITS1-1F-R: 5′-GCTGCGTTCTTCATCGATGC-3′ for ITS) tagged with abarcode. PCR was conducted in a total reaction volume of 30 μL containing 15 μL of Phusion® High-Fidelity PCR Master Mix (New England Biolabs), 0.2 μM forward and reverse primers, and approximately 10 ng of template DNA. The thermal cycling consisted of initial denaturation at 98°C for 1 min, followed by 30 denaturation cycles at 98°C for 10 s, annealing at 50°C for 30 s, elongation at 72°C for 60 s, and finally 72°C for 5 min. An equal volume of 1X loading buffer (containing SYBR green) was mixed with the PCR products and electrophoresed on 2% agarose gel for detection. Samples with bright main bands between 400 and 450 bp were chosen for further experiments. The PCR products were mixed in equidensity ratios and purified using the Gene JET Gel Extraction Kit (Thermo Scientific). Sequencing libraries were generated using the NEB Next® Ultra™ DNA Library Prep Kit for Illumina^®^ (NEB, USA) according to the manufacturer’s recommendations, and index codes were added. The quality of the library was assessed using the Qubit^®^ 2.0 Fluorometer (Thermo Scientific) and the Agilent 2100 Bioanalyzer system. The library was sequenced on an Illumina HiSeq 2500 platform by Beijing Novogene Bioinformatics Technology Co. Ltd. The raw sequencing data for 16S rRNA and ITS sequencing results have been deposited at the Sequence Read Archive (SRA, http://www.ncbi.nlm.nih.gov/sra/) under accession numbers PRJNA388067 (https://www.ncbi.nlm.nih.gov/bioproject/PRJNA388067) and PRJNA388081 (https://www.ncbi.nlm.nih.gov/bioproject/PRJNA388081), respectively.

### Processing of the sequencing data

Since the original raw data from the Illumina sequencing platform included some low-quality points, pre-processing was necessary for further analysis. The specific processing steps were as follows:

1. Data resolution: according to barcode sequences, each sample sequence was resolved from the raw data and the barcode sequences and primers were truncated;
2. Raw read splicing: the split data of each sample read were spliced using FLASH (V1.2.7, http://ccb.jhu.edu/software/FLASH/) for Mosaic [32], as it was the original tag data (raw tags);
3. Tag filtering: to obtain clean tags, the raw tags required stringent filter processing [33] according to the methods described by Caporaso et al. [34]. The raw tags were truncated at the first low quality base site when the continuous length of low-quality value (quality value ≤3) bases was up to three. The truncated tags in which continuous high quality base length was less than 75% of the tag lengths were excluded.
4. Chimera removal: the clean tags were contrasted with the Gold database (http://drive5.com/uchime/uchime_download.html) using the UCHIME Algorithm (http://www.drive5.com/usearch/manual/uchime_algo.html) to test the chimeric sequences. Final valid data (effective tags) were obtained after removing the chimeric sequences [35, 36]. Finally, the effective tags were used for subsequent analysis.

### Operational taxonomic units statistics and classification

To study species diversity, the effective tags were clustered using the UPARSE software [37]. Sequences with ≥97% similarity were assigned to the same operational taxonomic units (OTUs). Since the number of taxon tags was different for each of the six sequenced samples, the OTUs clustering results were standardized for subsequent analysis according to the minimum value of the sequence. The OTUs statistical information of the RS1 and RS2 samples was rendered using the Wayne figure.

Sequences in the same OTUs are considered as a corresponding taxon. In the process of assigning OTUs, the highest frequency sequence in the same OTUs was selected as the representative sequence. The representative sequences were used for classification. The 16S rRNA sequences were assigned with the Greengenes database using the RDP Classifier (threshold value is 0.8–1), and the ITS sequences were assigned with the UNITE/INSDC database using QIIME (threshold value is 0.8–1) [38–40].

### Microorganism community statistics and cluster analysis

After assigning the corresponding database, the OTUs were classified into different classification levels (i.e., Kingdom, Phylum, Class, Order, Family, Genus, and Species). The level into which more than 80% of the OTUs could be classified was chosen for microorganism community analysis. The relative microorganism abundance in each sample was determined according to the number of OTU reads.

To determine the difference in the microorganisms between the two groups, microorganism community cluster analysis was performed based on the Z value using the software MeV 4.9. The Z value of one sample in a classification was equal to the value calculated as the difference between the relative abundance in the sample and the average relative abundance in all samples, divided by the standard deviation of all samples.

## Results

### Symptoms of clubroot in the field

**Fig 1:**
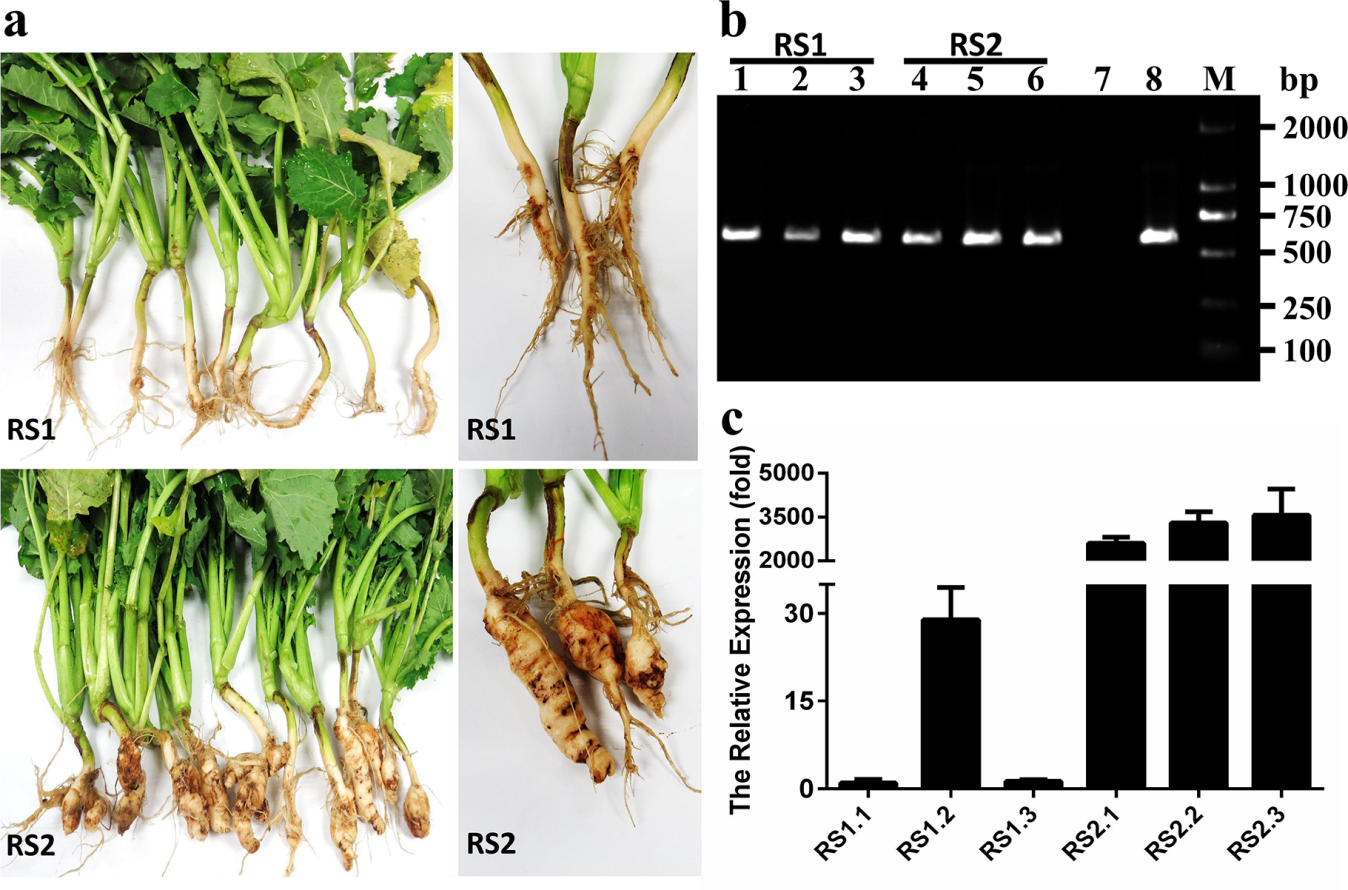
Symptoms of *Brassica napus* samples and PCR detection. **(a),** Symptoms of *B*. *napus* samples collected from the same clubroot field; photos of asymptomatic (above) and symptomatic (underneath) samples. **(b),** Detection of *P*. *brassicae* in surface-peeled roots of RS1 and RS2 samples; Lane 7: sterile water; Lane 8: *P*. *brassicae* DNA; Lane M: DNA marker. **(c),** q-PCR detection of *P*. *brassicae* in surface-peeled roots of RS1 and RS2 samples; The *B*. *napus* actin gene served as the internal control for normalization.

The symptoms of the clubroot samples are shown in **Fig 1**. The roots of RS1 samples were not obviously swollen and the lateral roots remained healthy, whereas the roots of RS2 samples were obviously swollen, and few lateral roots could be observed (**Fig 1A**). PCR amplification results showed that *P*. *brassicae* could be successfully detected in both RS1 and RS2 samples (**Fig 1B**). To evaluate the relative *P*. *brassicae* content in RS1 and RS2 samples, expression of the *P*. *brassicae* actin gene was measured using q-PCR. In the symptomatic (RS2) samples, the *P*. *brassicae* numbers were thousand-fold higher than those in the asymptomatic (RS1) samples **(Fig 1C).**

### Sequencing quality assessment

Raw data were generated by Illumina sequencing. Impurities such as joint and primer sequences, and low-quality reads were first removed to obtain effective tags for subsequent analyses. Sequencing quality was determined using the number of effective tags, their proportion in the raw data, and the base percentage of the sequencing error rate, which is less than 0.1% (Q30). In the case of 16S rRNA sequencing (**S1 Table**), 45471, 27897, 82715, 57064, 25939, and 20842 tags were generated as raw data for the six samples (RS1.1, RS1.2, RS1.3, RS2.1, RS2.2, and RS2.3, respectively). For each sample, the percentage of effective tags in the raw data was up to 92%, and the Q30 values were above 96%. In the case of ITS sequencing (**S2 Table**), 66344, 66782, 55796, 44365, 44576, and 66344 tags were generated as raw data for the six samples (RS1.1, RS1.2, RS1.3, RS2.1, RS2.2, and RS2.3, respectively). The percentage of effective tags in the raw data was up to 96% and the Q30 values were above 99% in each sample. This demonstrated that the data were suitable for subsequent analyses.

### OTU statistics and analysis

All effective tags from the six samples were clustered into OTUs according to sequence similarity (≥97%) using the UPARSE software (Edgar & Robert, 2013). According to the OTU statistical results, most effective tags (Taxon Tags) could be clustered to OTUs, with the exception of only a few data (Unclassified Tags and Unique Tags) (**S1 Fig**). The number of OTU clusters formed for the RS1 samples (RS1.1, RS1.2, and RS1.3) as a result of 16S rRNA and ITS sequencing were 114, 108, and 123, and 135, 133, and 148, respectively; and for the RS2 samples (RS2.1, RS2.2 and RS2.3) the number of OTU clusters formed were 93, 63, and 88, and 87, 81, and 69, respectively (**S1 Fig**).

**Fig 2:**
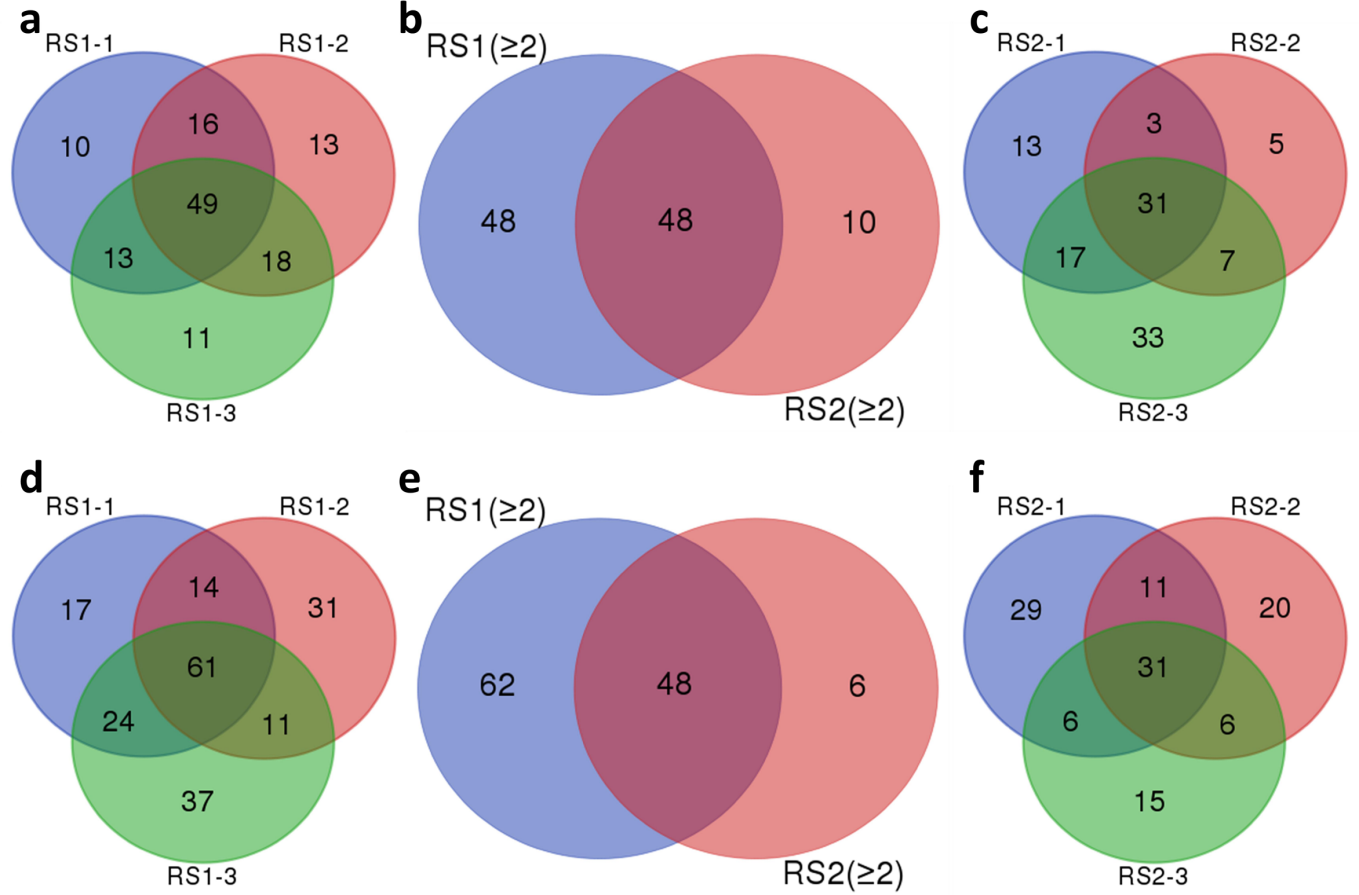
Number of operational taxonomic units (OTUs) in different samples. Venn diagrams were used to show the number of standardized OTUs in the three RS1 (asymptomatic) samples (RS1.1, RS1.2 and RS1.3) detected from 16S rRNA **(a)** and ITS **(d)** sequencing results, as well as the OTU numbers in the three RS2 (asymptomatic) samples (RS2.1, RS2.2 and RS2.3) detected from 16S rRNA **(c)** and ITS **(f)** sequencing results; The Venn diagrams were also used to express the number of OTUs present in at least two samples from each group between the RS1 (asymptomatic) samples (RS1 ≥2) and the RS2 (asymptomatic) samples (RS2 ≥2) detected from 16S rRNA **(b)** and ITS **(e)** sequencing results.

Since the number of taxon tags in each sequencing sample was different, the OTU clusters formed above could not be directly used tocompare the two samples. The OTU clustering results were standardized for subsequent analysis on the basis of the minimal sequence read number. The standardized OTU information for RS1 and RS2 samples was rendered using the Wayne figure (**Fig 2**). In both RS1 and RS2 groups, three samples were used for sequencing. Shared and unique OTUs were found among the three samples. To ensure the accuracy and completeness of the data, OTUs that existed in at least two of the three samples were chosen for further study. In the RS1 group, 96 OTUs (49 shared and 47 in two samples) from 16S rRNA and 110 OTUs (61 shared and 49 in two samples) from ITS were used for subsequent studies (**Fig 2A and 2D**). In the RS2 group, 58 OTUs (31 shared and 27 in two samples) from 16S rRNA and 54 OTUs (31 shared and 23 in two samples) from ITS were used for further studies (**Fig 2C and 2F**). Comparison of the OTUs in at least two samples from each group showed that, in 16S rRNA 48 OTUs were common to both groups, 48 OTUs were only in the group RS1, and 10 OTUs were only in the group RS2 (**Fig 2B**). In ITS, 48 OTUs were common to both groups, 62 OTUs existed only in the group RS1, and 6 OTUs were only in the group RS2 (**Fig 2D**). Therefore, 106 OTUs from 16S rRNA and 116 OTUs from ITS were used for further analysis. The number of OTUs in the group RS1 was obviously greater than that in the group RS2, both from 16S rRNA and ITS sequencing results.

### OTU classification and microorganism community analysis

After the OTU cluster analysis, the same OTU sequences were assumed to derive from a certain taxon, and a representative sequence for each OTU was classified into different classification levels (Kingdom, Phylum, Class, Order, Family, Genus, and Species).[38, 39]. The OTU classification information and statistics results are shown in **S3 and S4 Tables and S2 Fig**. More than 80% of the OTUs could be classified at the family level from the 16S rRNA sequencing results (**S2A Fig**) and at the genus level from the ITS sequencing results (**S2B Fig**); thus, the family and genus taxa were used to analyze the bacterial and fungal communities. The 106 OTUs from 16S rRNA and 116 OTUs from ITS as obtained above were classified into 45 bacterial families and 71 fungal genera (**S3 and S4 Tables**). To determine the differences in endosphere microorganism communities between the asymptomatic (RS1) and symptomatic (RS2) *B*. *napus* samples, the relative abundance of bacteria and fungi in each sample was calculated based on the number of OTU reads and used for analysis **(S3 and S4 Tables**).

**Fig 3:**
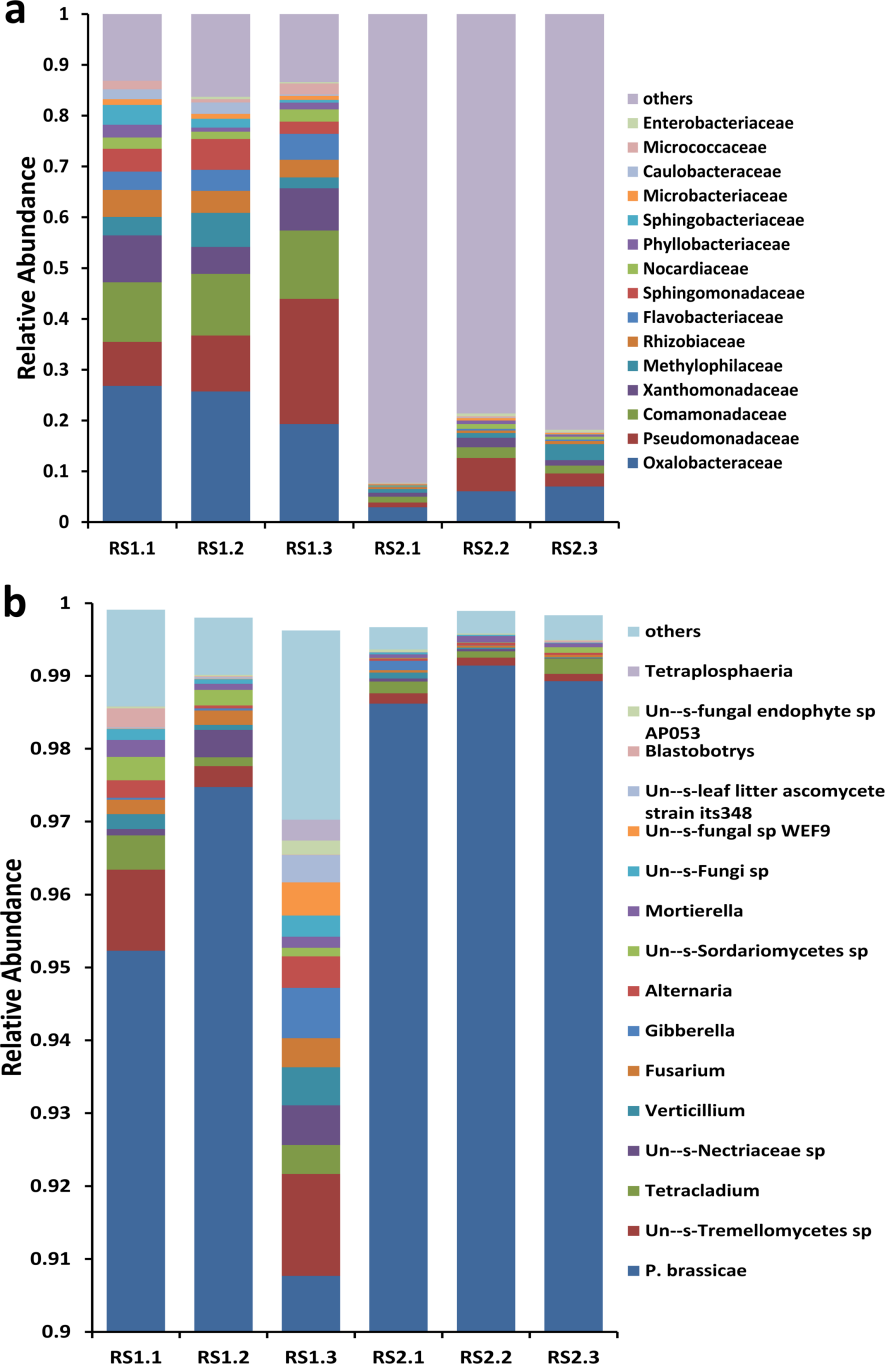
**Relative microbial abundance from 16S rRNA sequencing results at the family level (a) and ITS sequencing results at the genus level (b). (a),** Relative abundance of the top 15 bacterial families from the 16S rRNA sequencing results; **(b),** Relative abundance of the top 16 fungal genera from the ITS sequencing results.

Based on the classification results, 39 and 28 bacterial families were found in the asymptomatic RS1 and symptomatic RS2 groups, respectively (**S3 Table**). The two most abundant families (i.e., Streptophyta and Mitochondria) were excluded from the analysis. Except these two families, the relative abundances of the top 15 bacterial families in each sample is shown in **Fig 3A**. The most abundant family was Oxalobacteraceae, followed by Pseudomonadaceae, Comamonadaceae, Xanthomonadaceae, Methylophilaceae, Rhizobiaceae, Flavobacteriaceae. The relative abundance of these bacteria in the asymptomatic samples (RS1.1, RS1.1 and RS1.3) was much greater than that in the symptomatic samples (RS2.1, RS2.1 and RS2.3) (**Fig 3A, S3 Table**). In symptomatic samples, the relative abundance of other bacteria was the highest (0.92, 0.78, and 0.82), whereas in asymptomatic samples, the relative abundance of other bacteria was low (0.13, 0.16, and 0.13).

The ITS sequencing results indicated the endosphere fungal communities and *P*. *brassicae*. The most abundant species in all six samples was undoubtedly *P*. *brassicae*, with its content in the symptomatic samples (0.986, 0.991, and 0.989) was slightly higher than that in the asymptomatic samples (0.952, 0.975, and 0.908). Based on the classification, 65 and 39 fungal genera were found in RS1 and RS2 groups, respectively (**S4 Table**). The most abundant fungal genera were Un—s-Tremellomycetes sp., *Tetracladium*, Un—s-Nectriaceae sp., *Verticillium*, *Fusarium*, *Gibberella*, *Alternaria*, Un—s-Sordariomycetes sp., and *Mortierella*. The relative abundance of these fungi in the asymptomatic samples was also much greater than that in the symptomatic samples (**Fig 3B**). In the asymptomatic samples, other fungi (0.013, 0.007, and 0.026) were present in greater amounts than those in the symptomatic samples (0.003, 0.003, and 0.003).

### Microorganism community clustering and specific microorganism analysis

**Fig 4:**
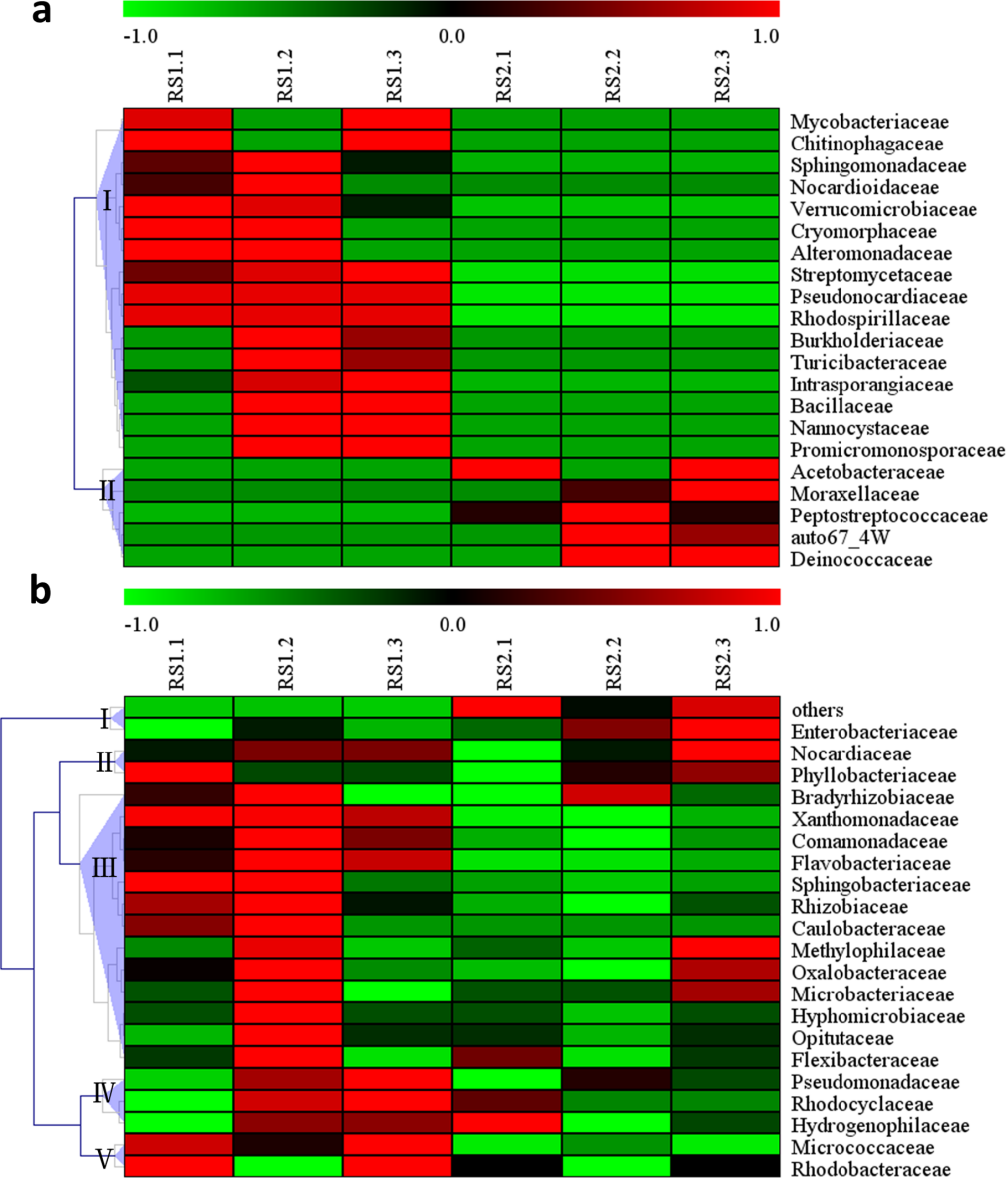
**Abundance of clusters from 16S rRNA with unique families (a) and shared families (b).** The 16S rRNA sequencing results revealed **(a)** 21 unique families (16 families in the asymptomatic samples and five families in the symptomatic samples) and **(b)** 21 families that were common to both the asymptomatic and symptomatic samples. The clusters were based on the Z value. The Z value of one sample in a classification is equal to the value calculated from the difference between the relative abundance in the sample and the average relative abundance in all samples, divided by the standard deviation of all samples.

For further analysis of the differences in the endosphere communities between the asymptomatic and symptomatic samples, the relative abundance of all bacterial families and fungal genera was clustered. For the bacterial endosphere community, 16 families existed only in the asymptomatic samples, five families only in the symptomatic samples, and 21 families common to both samples (**Fig 4**). Among the 16 families that were present only in the asymptomatic samples, Streptomycetaceae, Pseudonocardiaceae, Rhodospirillaceae, Sphingomonadaceae, and Verrucomicrobiaceae were present in all three groups (**Fig 4AI**), and the remaining families (i.e., Intrasporangiaceae, Nocardioidaceae, Mycobacteriaceae, Cryomorphaceae, Burkholderiaceae, Turicibacteraceae, Alteromonadaceae, Bacillaceae, Chitinophagaceae, Nannocystaceae, and Promicromonosporaceae) were present in two of the three samples. The families present only in the symptomatic samples included Peptostreptococcaceae, Moraxellaceae, Acetobacteraceae, Deinococcaceae, and an unclassified bacterium (auto67_4W) (**Fig 4AII**).

Of the 21 shared families, the relative abundance of Nocardiaceae, Phyllobacteriaceae, and Enterobacteriaceae in the symptomatic samples was greater than that in the asymptomatic samples (**Fig 4BI and 4BII**). The abundance of Methylophilaceae, Oxalobacteraceae, and Microbacteriaceae was similar in all the samples (**Fig 4BIII**). The remaining 15 families were more abundant in the asymptomatic samples than in the symptomatic samples, especially Xanthomonadaceae, Comamonadaceae, Flavobacteriaceae, Sphingobacteriaceae, Rhizobiaceae, Caulobacteraceae, and Bradyrhizobiaceae (**Fig 4BIII, 4BIV and 4BV**).

**Fig 5:**
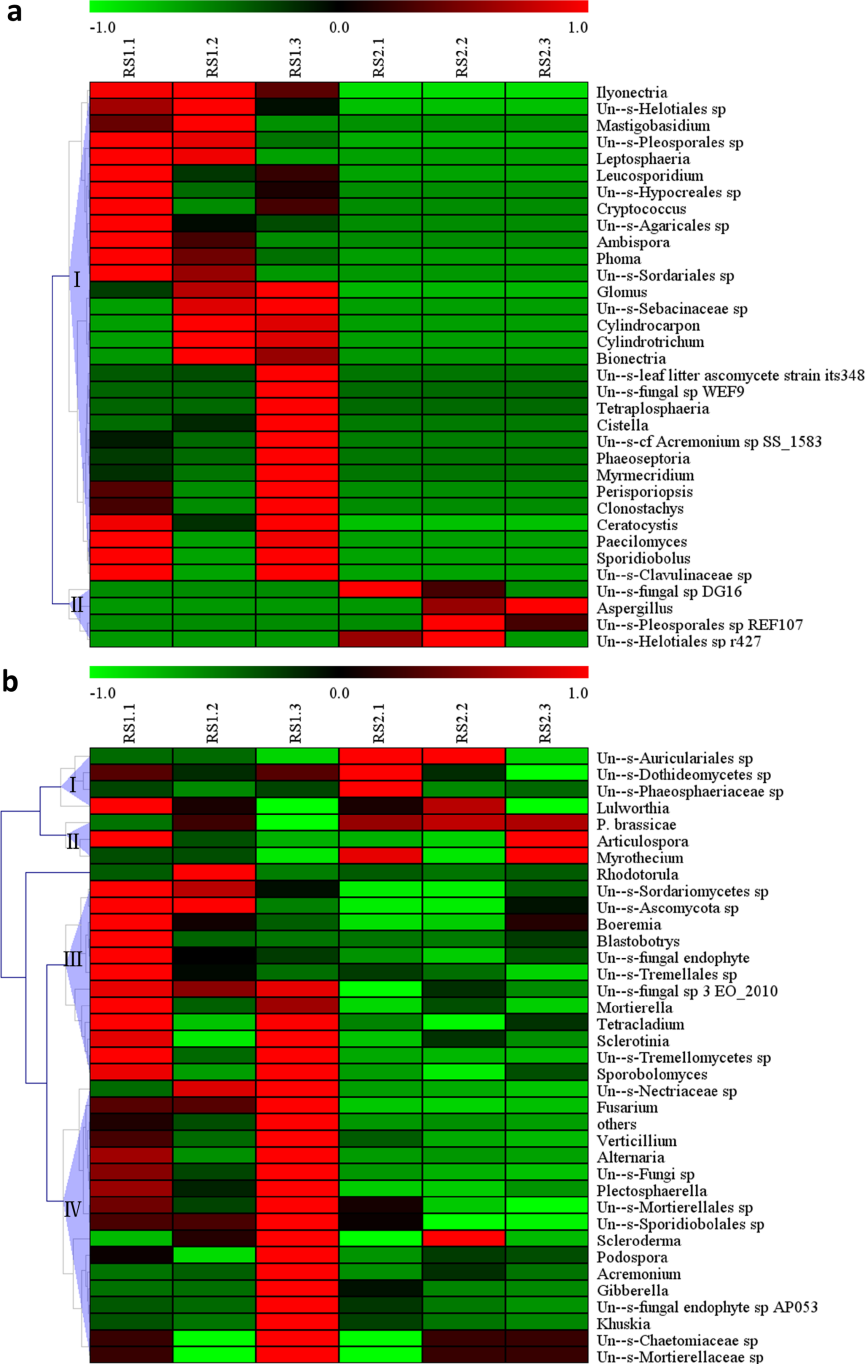
**Abundance of clusters from ITS with unique families (a) and shared families (b).** The ITS sequencing results revealed **(a)** 34 unique families (30 families in the asymptomatic samples and four families in the symptomatic samples) and **(b) **37 families shared in both the asymptomatic and symptomatic samples. The clusters were based on the Z value. The Z value of one sample in a classification was equal to the value calculated for the difference between the relative abundance in the sample and the average relative abundance in all samples, divided by the standard deviation of all samples.

In the fungal endosphere community, 30 genera were unique to the asymptomatic samples, including *Tetraplosphaeria*, *Phaeoseptoria*, *Cistella*, *Phoma*, *Ilyonectria*, *Glomus*, *Ambispora*, and Un—s-Hypocreales sp. (**Fig 5AI**). Only four genera were detected in the symptomatic samples, namely Un—s-Pleosporales sp. REF107, *Aspergillus*, Un—s-fungal sp. DG16, and Un—s-Helotiales sp. r427 (**Fig 5AII**). Among the 37 shared genera, seven, including *P*. *brassicae*, were much more abundant in the symptomatic samples than in the asymptomatic samples (**Fig 5BI and 5BII**). The abundance of the remaining 30 genera including *Tetracladium*, *Fusarium*, *Alternaria*, Un—s-fungal sp. 3 EO_2010, and *Sporobolomyces* was much greater in the asymptomatic samples than in the symptomatic samples (**Fig 5BIII and 5BIV**).

Overall, the bacterial and fungal endosphere communities in the asymptomatic samples were more abundant than those in the symptomatic samples. Among the unique and highly abundant bacteria and fungi in the asymptomatic samples, six bacterial families and six fungal genera have been attributed with biological control functions, three bacterial families with plant growth activity, and four bacterial families and seven fungal genera causing plant disease. Interestingly, Rhizobiaceae and Bradyrhizobiaceae were detected in both the asymptomatic and symptomatic samples, and their relative abundance in the asymptomatic samples was much greater than that in the symptomatic samples. Furthermore, *Glomus* was detected only in the asymptomatic samples, whereas *Aspergillus* was detected only in the symptomatic samples (**Tables 1 and 2**).

**Table 1:** Bacterial classification statistics based on function.

Function	Bacteria		
	Family Name	Relative content	
		RS1 (Avg)	RS2 (Avg)
**Biological** **Control**	Streptomycetaceae^[^^50^^]^	2.08E−04	0
	Sphingomonadaceae^[^^52^^]^	1.09E−03	0
	Intrasporangiaceae^[^^52^^]^	1.91E−04	0
	Bacillaceae^[^^47^^]^	3.47E−05	0
	Flavobacteriaceae^[^^51^^]^	1.01E−03	1.91E−04
	Pseudomonadaceae^[^^52^^]^	3.33E−03	2.11E−03
**Plant Growth Promotion**	Burkholderiaceae^[^^54^^]^	5.20E−05	0
	Chitinophagaceae^[^^53^^]^	3.47E−05	0
	Nannocystaceae^[^^47^^]^	3.47E−05	0
**Disease Inducing**	Enterobacteriaceae^[^^19^^]^	6.93E−05	2.77E−04
	Xanthomonadaceae	1.68E−03	9.01E−04
	Mycobacteriaceae	8.67E−05	0
	Micrococcaceae	3.12E−04	6.93E−05
**Others**	Rhizobiaceae	1.02E−03	3.99E−04
	Bradyrhizobiaceae	1.04E−04	6.93E−05

**Table 2:** Fungal classification statistics based on function.

Function	Fungi		
	Genus Name	Relative content	
		RS1 (Avg)	RS2 (Avg)
**Biological** **Control**	*Rhodotorula*^[^^55^^]^	6.38E−04	9.33E−05
	*Sporobolomyces*^[^^55^^]^	1.17E−04	2.33E−05
	*Sporidiobolus*^[^^56^^]^	8.55E−05	0
	*Leucosporidium*^[^^57^^]^	7.00E−05	0
	*Un—s-Sporidiobolales* sp.^[^^56^^]^	1.79E−04	4.67E−05
	*Un—s-Hypocreales* sp.	3.89E−04	0
**Disease Inducing**	*Fusarium*	2.65E−03	3.19E−04
	*Gibberella*	2.49E−03	4.43E−04
	*Alternaria*^[^^1^^]^	2.37E−03	3.19E−04
	*Sclerotinia*	5.29E−04	2.02E−04
	*Phoma*	1.63E−04	0
	*Leptosphaeria*	9.33E−05	0
	*Cylindrocarpon*	3.89E−05	0
**Others**	*Glomus*	7.00E−05	0
	*Aspergillus*	0	4.67E−05

## Discussion

Recently, research on microbiome has attracted increasing attention. Previously, 16S rRNA and ITS sequencing have been widely used in plant microbiome studies [35–42]. In this study, we analyzed the root endosphere microbiome of *B*. *napus* infested with *P*. *brassicae* using 16S rRNA and ITS sequencing, and tailored the sequencing quality to the requirement of the analysis. The 16S rRNA sequencing results revealed 106 OTUs (OTUs that existed in at least two of the three samples), and 116 OTUs were detected from the ITS sequencing results. The majority of OTUs (except OTU3) from the 16S rRNA sequencing results could be classified to the family level and most OTUs from the ITS sequencing results could be classified to the genus level (**S2 Fig**).

Although the results showed that 16S rRNA and ITS sequencing work well in microbiome research, some difficulties persist. For instance, in the 16S rRNA sequencing results, 11, 15, and 10 OTUs in the asymptomatic samples (RS1.1, RS1.2, and RS1.3) and 9, 6, and 7 OTUs in the symptomatic samples (RS2.1, RS2.2, and RS2.3) could not be classified into any known family. In the symptomatic samples, OTU 3 was classified as an uncultured bacterial clone and its relative content was 70–90% of all the OTUs. The ITS sequencing results also showed 18, 23, and 25 OTUs in the asymptomatic samples (RS1.1, RS1.2 and RS1.3) and 14, 8, and 8 OTUs in the symptomatic samples (RS2.1, RS2.2 and RS2.3) that could not be classified into any known genera (**S2 Fig**). This might suggest that new microorganisms were being hosted by *P*. *brassicae*-infected *B*. *napus* roots including unknown bacteria (i.e., OTU 3). There may be two possible reasons for this. First, our knowledge of microorganisms is still very limited especially for those that cannot be purified and cultivated. Secondly, only 500 bp sequences were created and analyzed in the 16S rRNA sequencing platform, which is a little short for bacterial identification. Moreover, it is difficult to overcome the chloroplast and mitochondrial sequence interference in 16S rRNA sequencing [43]. Moreover, since fungal ITS sequences in the public database are relatively poor compared to the bacterial 16S rRNA gene sequences, it is necessary to include other genes such as EF-1α, β-tubulin, and RNA polymerases to complete the identification [44]. Therefore, achieving a comprehensive understanding of the microbiome and its functions will take a long time.

The main purpose of this study was to clarify the difference between the endosphere microbiomes of symptomatic and asymptomatic *B*. *napus* roots from the same natural clubroot field, using 16S rRNA and ITS sequencing. Based on the results, we first found that *P*. *brassicae* was common in both the symptomatic and asymptomatic samples. Second, many bacterial families and fungal genera were unique to the asymptomatic samples. Moreover, most of the shared bacteria and fungi present across both groups were more abundant in the asymptomatic samples. These results were similar to those of previous studies. *Pseudomonas syringae* pv. *actinidiae* was detected in both the symptomatic and asymptomatic tissues of kiwifruit [45]. The microbial population balance (mainly *Methylobacterium* spp., *C*. *flaccumfaciens*, and *X*. *fastidiosa*) was also related to the CVC asymptomatic or symptomatic presentation in citrus plants [20]. Besides, in previous studies, some beneficial microorganisms were found to be much more salient in asymptomatic plants. For example, numerous plant-beneficial bacterial isolates were found in the asymptomatic plants of Huanglongbing diseased citrus compared to the symptomatic plants [21]; *C*. *flaccumfaciens* abundance in asymptomatic plants was higher than that in symptomatic plants, and may contribute to CVC resistance [19]. In our study, many of the unique and highly abundant bacteria and fungi in the asymptomatic samples have been attributed with biological control or plant growth promotion functions. *Bacillus* sp. from the family Bacillaceae, was reported as a biocontrol agent for *P*. *brassicae* [44, 46, 47]; *Streptomyces* spp. [48, 49] and *Streptomyces griseoruber* A316 [50] from the family Streptomycetaceae have been reported to reduce the severity of clubroot disease; *Flavobacteriumhercynim* EPB-C313, an endophytic bacteria of the family Flavobacteriaceae, has been used to control Chinese cabbage clubroot [51]. In this study, we identified 1, 1, and 7 OTUs in the asymptomatic samples belonging to Bacillaceae, Streptomycetaceae, and Flavobacterium families, respectively. Strains of Sphingomonadaceae can inhibit *Pythium spinosum*, whereas strains of Pseudomonadaceae efficiently control fusarium wilt disease [14, 15, 52]. Species from the Chitinophagaceae and Burkholderiaceae families can promote plant growth [53, 54]. Yeasts from *Sporobolomyces*, *Leucosporidium*, and *Sporidiobolus* can be used to control gray mold [55–57]. In asymptomatic samples, 9 OTUs were detected as belonging to the six above-mentioned families. They may have similar effects on oilseed rape and clubroot disease. Above all, we assume that the differences in the endosphere microbiome of the symptomatic and asymptomatic samples in the endosphere microbiome may influence the development of clubroot symptoms.

Another purpose of this study was to exploit the endosphere microbiome analysis technique to mine microorganism resources for resistance to clubroot disease. Little is known about Rhodocyclaceae, Verrucomicrobiaceae, Pseudonocardiaceae, and “Un—s-” fungi, all of which were only present in the asymptomatic samples, along with the unknown bacteria (OUT 3) in the symptomatic samples. These microorganisms may be potential bio-control resources for preventing clubroot disease. Moreover, in the asymptomatic *B*. *napus* root samples, a variety of pathogenic plant microorganisms from other crops were detected including Xanthomonadaceae, *Fusarium*, *Gibberella*, *Alternaria*, *Sclerotinia*, *Phoma*, *Leptosphaeria*, and *Cylindrocarpon*. The impact of these pathogens on non-host plants remains a topic for further study.

A few anaerobic or facultative anaerobic bacteria like Acetobacteraceae [58] were unique to symptomatic *B*. *napus* root samples, suggesting that *P*. *brassicae* might consume oxygen or need environmental hypoxia when growing in *B*. *napus* roots [59]. Interestingly, some fungi from the genus *Aspergillus* are typical saprophytes or opportunistic pathogens [60], and *Aspergillus* could only be detected in the symptomatic samples. Therefore, we postulate that *P*. *brassicae* needs to decay and disintegrate the host roots with the help of saprophytic microorganisms, in order to release its resting spores into the soil at the later stages of clubroot development. Furthermore, we also found an interesting phenomenon that bacteria from the family Rhizobiaceae and Bradyrhizobiaceae and fungi from the genus *Glomus* were detected *in B*. *napus* infested with *P*. *brassicae*, although *B*. *napus* is not a plant that hosts rhizobia and arbuscular mycorrhizal fungi. Further analysis on this phenomenon is underway.

In summary, this study was mainly conducted to compare the differences in the endosphere microbiomes of symptomatic and asymptomatic *B*. *napus* roots collected from the same natural clubroot field using 16S rRNA and ITS sequencing. The microbe population and their relative abundance in asymptomatic roots are far greater than that in the symptomatic *B*. *napus* roots. Many microorganisms detected in the asymptomatic roots have biological control and plant growth promotion functions. Furthermore, we also found many pathogenic plant microorganisms to which *B*. *napus* is not a host, and the related mechanism underlying this observation needs further research. These results provide a new basis for studying the endosphere microbiomes of roots and some potential bio-control resources for clubroot prevention.

## Supporting information

S1 Fig The number of Tags and OTUs in different samples. The statistical figures show the number of Tags and OTUs for all samples from the 16S rRNA sequencing results **(a)** and ITS sequencing results **(b)**. Total Tags, the filtered splicing sequence numbers; Taxon Tags, the number of tags used to build the OTUs and gain classification information; Unclassified Tags, the number of tags used to build OTUs but which did not provide any classification information; Unique Tags, the number of tags for which the frequency is one and cannot be clustered into OTUs; OTUs, the final OTU numbers.
(TIF)

S2 Fig The number of OTUs at each sample classification level. **(a),** The OTUs were classified by the taxonomic information into different bacterial classification levels detected in the 16S rRNA sequencing results. The above row of numbers refers to the number of OTUs classified to the bacterial Kingdom level and the lower row of numbers refers to the number of OTUs classified to the bacterial Family level. **(b),** The OTUs were classified by the taxonomic information into different fungal classification levels detected in the ITS sequencing results. The above row of numbers refers to the number of OTUs classified into the fungal Kingdom level and the lower row of numbers refers to the number of OTUs classified into the fungal Genus level.
(TIF)

S1 Table Data preprocessing statistics and quality analysis of rapeseed involving 16S rRNA sequencing of the root. Raw data, the number of PE reads; Raw Tags, Tag number of patchwork sequence; Clean Tags: Tags taken off the low quality tag number; Effective Tags, Tag number for aftershock; Base, The number of basesof the Effective Data; AvgLen, The average length of the Effective Tags; Q30, Base percentage of the sequencing error rate is less than 0.1% in Effective Tags; Effective (%), Effective Tags/PE Reads.
(DOCX)

S2 Table Data preprocessing statistics and quality analysis of rapeseed involving ITS sequencing of the root. Raw data: the number of PE reads; Raw Tags: Tag number of patchwork sequence; Clean Tags: Tags taken off thelow quality tag number; Effective Tags: Tag number for aftershock; Base: The number of bases of the Effective Data; AvgLen: The average length of the Effective Tags; Q30: Base percentage of the sequencing error rate is less than 0.1%in Effective Tags; Effective (%): Effective Tags/PE Reads.
(DOCX)

S3 Table Classification information and relative abundance of each OTU in the 16S rRNAsequencing results. (DOCX)

S4 Table Classification information and relative abundance of each OTU in the ITS sequencing results. (DOCX)

## References

[1] Lebeis SL, Rott M, Dangl JL, Schulze-Lefert. Culturing a plant microbiome community at the cross-Rhodes. New Phytol 2012; 196: 341–344. doi: 10.1111/j.1469-8137.2012.04336.x 22978611 10.1111/j.1469-8137.2012.04336.x

[2] Bulgarelli D, Schlaeppi K, Spaepen S, Ver Loren van Themaat E, Schulze-Lefert P. Structure and functions of the bacterial microbiota of plants. Annu Rev Plant Biol 2013; 64: 807–838. doi: 10.1146/annurev-arplant-050312-120106 23373698 10.1146/annurev-arplant-050312-120106

[3] Turner TR, James EK, Poole PS. The plant microbiome. Genome Biol 2013; 14: 209 doi: 10.1186/gb-2013-14-6-209 23805896 10.1186/gb-2013-14-6-209 PMC3706808

[4] Vandenkoornhuyse P, Quaiser A, Duhamel M, Le Van A, Dufresne A. The importance of the microbiome of the plant holobiont. New Phytol 2015; 206: 1196–1206. doi: 10.1111/nph.13312 25655016 10.1111/nph.13312

[5] Berendsen RL, Pieterse CM, Bakker PA. The rhizosphere microbiome and plant health. Trends Plant Sci 2012; 17: 478–486. doi: 10.1016/j.tplants.2012.04.001 22564542 10.1016/j.tplants.2012.04.001

[6] Mendes R, Kruijt M, de Bruijn I, Dekkers E, van der Voort M, Schneider JH, et al Deciphering the rhizosphere microbiome for disease-suppressive bacteria. Science 2011; 332: 1097–1100. doi: 10.1126/science.1203980 21551032 10.1126/science.1203980

[7] Bais HP, Weir TL, Perry LG, Gilroy S, Vivanco JM. The role of root exudates in rhizosphere interactions with plants and other organisms. Annu Rev Plant Biol 2006; 57: 233–266. doi: 10.1146/annurev.arplant.57.032905.105159 16669762 10.1146/annurev.arplant.57.032905.105159

[8] van der Heijden MGA, Bardgett RD, van Straalen NM. The unseen majority: soil microbes as drivers of plant diversity and productivity in terrestrial ecosystems. Ecol Lett 2008; 11: 296–310. doi: 10.1111/j.1461-0248.2007.01139.x 18047587 10.1111/j.1461-0248.2007.01139.x

[9] Provorov NA, Vorobyov NI. Host plant as an organizer of microbial evolution in the beneficial symbioses. Phytochem Rev 2009; 8: 519–534.

[10] Schüßler A, Schwarzott D, Walker C. A new fungal phylum, the Glomeromycota: phylogeny and evolution. Mycol Res 2001; 105: 1413–1421.

[11] Casieri L, Lahmidi NA, Doidy J, Veneault-Fourrey C, Migeon A, Bonneau L, et al Biotrophic transportome in mutualistic plant-fungal interactions. Mycorrhiza 2013; 23: 597–625. doi: 10.1007/s00572-013-0496-9 23572325 10.1007/s00572-013-0496-9

[12] Freeman BC, Chen C, Beattie GA. Identification of the trehalose biosynthetic loci of *Pseudomonas syringae* and their contribution to fitness in the phyllosphere. Environ Microbial 2010; 12: 1486–1497. 10.1111/j.1462-2920.2010.02171.x 20192963

[13] Chen L, Huang X, Zhang F, Zhao D, Yang X, Shen QR. Application of *Trichoderma harzianum* SQR-T037 bio-organic fertilizer significantly controls *Fusarium* wilt and affects the microbial communities of continuously cropped soil of cucumber. J Sci Food Agric 2012; 92: 2465–2470. doi: 10.1002/jsfa.5653 22513876 10.1002/jsfa.5653

[14] Brooks DS, Gonzalez CF, Appel DN, Filer TH. Evaluation of endophytic bacteria as potential biological-control agents for Oak Wilt. Biol Control 1994; 4:373–381.

[15] Chen C, Bauske EM, Musson G, Rodriguezkabana R, Kloepper J. Biological control of *Fusarium* wilt on cotton by use of endophytic bacteria. Biol Control 1995; 5: 83–91.

[16] Xiao K, Kinkel LL, Samac DA. Biological control of Phytophthora root rots on alfalfa and soybean with Streptomyces. Biol Control 2002; 23: 285–295.

[17] Hwang SF, Strelkov SE, Feng J, Gossen BD, Howard RJ. *Plasmodiophora brassicae*: a review of an emerging pathogen of the Canadian canola (*Brassica napus*) crop. Mol Plant Pathol 2012; 13: 105–113. doi: 10.1111/j.1364-3703.2011.00729.x 21726396 10.1111/j.1364-3703.2011.00729.x PMC6638701

[18] Chen T, Bi K, He Z, Gao Z, Zhao Y, Fu Y, et al *Arabidopsis* Mutant bik1 exhibits strong resistance to *Plasmodiophora brassicae*. Front Physiol 2016; 7: 402–415. doi: 10.3389/fphys.2016.00402 27679580 10.3389/fphys.2016.00402 PMC5020103

[19] Araújo WL, Marcon J, Maccheroni W, van Elsas JD, van Vuurde JW, Azevedo JL. Diversity of endophytic bacterial populations and their interaction with *Xylella fastidiosa* in citrus plants. Appl Environ Microbiol 2002; 68: 4906–4914. doi: 10.1128/AEM.68.10.4906-4914.2002 12324338 10.1128/AEM.68.10.4906-4914.2002 PMC126398

[20] Lacava PT, Araújo WL, Marcon J, Maccheroni W, Azevedo JL. Interaction between endophytic bacteria from citrus plants and the phytopathogenic bacteria *Xylella fastidiosa*, causal agent of citrus-variegated chlorosis. Lett Appl Microbiol 2004; 39: 55–59. doi: 10.1111/j.1472-765X.2004.01543.x 15189288 10.1111/j.1472-765X.2004.01543.x

[21] Trivedi P, Spann T, Wang N. Isolation and characterization of beneficial bacteria associated with citrus roots in Florida. Microbial Ecol 2011; 62: 324–336. 10.1007/s00248-011-9822-y 21360139

[22] Bruez E. Etude Comparative des Communautés Fongiques et Bactériennes Colonisant le Bois De Ceps De Vigne Ayant Exprimé Ou Non Des Symptômes D’esca Ph.D. thesis, University of Bordeaux, Bordeaux, 2013; 258.

[23] Bachy C, Dolan JR, López-García P, Deschamps P, Moreira D. Accuracy of protist diversity assessments: morphology compared with cloning and direct pyrosequencing of 18S rRNA genes and ITS regions using the conspicuous tintinnid ciliates as a case study. ISME J 2012; 7: 244–255. doi: 10.1038/ismej.2012.106 23038176 10.1038/ismej.2012.106 PMC3554406

[24] Youssef N, Sheik CS, Krumholz LR, Najar FZ, Roe BA, Elshahed MS. Comparison of species richness estimates obtained using nearly complete fragments and simulated pyrosequencing-generated fragments in 16S rRNA gene-based environmental surveys. Appl Environ Microbiol 2009; 75: 5227–5236. doi: 10.1128/AEM.00592-09 19561178 10.1128/AEM.00592-09 PMC2725448

[25] Caporaso JG, Lauber CL, Walters WA, Berg-Lyons D, Lozupone CA, Turnbaugh PJ, et al Global patterns of 16S rRNA diversity at a depth of millions of sequences per sample. Proc Natl Acad Sci USA 2011; 108(Supplement 1): 4516–4522. 20534432 10.1073/pnas.1000080107 PMC3063599

[26] Hess M, Sczyrba A, Egan R, Kim TW, Chokhawala H, Schroth G, et al Metagenomic discovery of biomass-degrading genes and genomes from cow rumen. Science 2011; 331: 463–467. doi: 10.1126/science.1200387 21273488 10.1126/science.1200387

[27] Degnan PH, Howard O. Illumina-based analysis of microbial community diversity. ISME J 2012; 6: 183–194. doi: 10.1038/ismej.2011.74 21677692 10.1038/ismej.2011.74 PMC3246231

[28] Berg G, Grube M, Schloter M, Smalla K. The plant microbiome and its importance for plant and human health. Front Microbiol 2014; 5: 1. 25278934 10.3389/fmicb.2014.00491 PMC4166366

[29] Hardoim PR, Van Overbeek LS, Berg G, Pirttilä AM, Compant S, Campisano A, et al The hidden world within plants: ecological and evolutionary considerations for defining functioning of microbial endophytes. Microbiol Mol Biol R 2015; 79: 293–320. 10.1128/MMBR.00050-14 PMC4488371 26136581

[30] Doyle JJ. Isolation of plant DNA from fresh tissue. Focus 1990; 12: 13–15.

[31] Wallenhammar A, Arwidsson O. Detection of *Plasmodiophora brassicae* by PCR in naturally infested soils. Eur J Plant Pathol 2001; 107: 313–321.

[32] Magoč T, Steven SL. FLASH: fast length adjustment of short reads to improve genome assemblies. Bioinformatics 2011; 27: 2957–2963. doi: 10.1093/bioinformatics/btr507 21903629 10.1093/bioinformatics/btr507 PMC3198573

[33] Bokulich NA, Subramanian S, Faith JJ, Gevers D, Gordon JI, Knight R, et al Quality-filtering vastly improves diversity estimates from Illumina amplicon sequencing. Nat Methods 2013; 10: 57–59. doi: 10.1038/nmeth.2276 23202435 10.1038/nmeth.2276 PMC3531572

[34] Caporaso JG, Kuczynski J, Stombaugh J, Bittinger K, Bushman FD, Costello EK, et al QIIME allows analysis of high-throughput community sequencing data. Nat Methods 2010; 7: 335–336. doi: 10.1038/nmeth.f.303 20383131 10.1038/nmeth.f.303 PMC3156573

[35] Edgar RC, Haas BJ, Clemente JC, Quince C, Knight R. UCHIME improves sensitivity and speed of chimera detection. Bioinformatics 2011; 27: 2194–2200. doi: 10.1093/bioinformatics/btr381 21700674 10.1093/bioinformatics/btr381 PMC3150044

[36] Haas BJ, Haas BJ, Gevers D, Earl AM, Feldgarden M, Ward DV, et al Chimeric 16S rRNA sequence formation and detection in Sanger and 454-pyrosequenced PCR amplicons. Genome Res 2011; 21: 494–504. doi: 10.1101/gr.112730.110 21212162 10.1101/gr.112730.110 PMC3044863

[37] Edgar RC. UPARSE: highly accurate OTU sequences from microbial amplicon reads. Nat Methods 2013; 10: 996–998. doi: 10.1038/nmeth.2604 23955772 10.1038/nmeth.2604

[38] DeSantis TZ, Hugenholtz P, Larsen N, Rojas M, Brodie EL, Keller K, et al Greengenes, a chimera-checked 16S rRNA gene database and workbench compatible with ARB. Appl Environ Microbiol 2006; 72: 5069–5072. doi: 10.1128/AEM.03006-05 16820507 10.1128/AEM.03006-05 PMC1489311

[39] Wang Q, Garrity GM, Tiedje JM, Cole JR. Naive Bayesian classifier for rapid assignment of rRNA sequences into the new bacterial taxonomy. Appl Environ Microbiol 2007; 73: 5261–5267. doi: 10.1128/AEM.00062-07 17586664 10.1128/AEM.00062-07 PMC1950982

[40] Kõljalg U, Nilsson RH, Abarenkov K, Tedersoo L, Taylor AF, Bahram M, et al Towards a unified paradigm for sequence-based identification of fungi. Mol Ecol 2013; 22: 5271–5277. doi: 10.1111/mec.12481 24112409 10.1111/mec.12481

[41] Balajee SA, Borman AM, Brandt ME, Cano J, Cuenca-Estrella M, Dannaoui E, et al Sequence-based identification of *Aspergillus*, *Fusarium*, and *Mucorales* species in the clinical mycology laboratory: where are we and where should we go from here? J Clin Microbiol 2009; 47: 877–884. doi: 10.1128/JCM.01685-08 19073865 10.1128/JCM.01685-08 PMC2668331

[42] Hanshew AS, Mason CJ, Raffa KF, Currie CR. Minimization of chloroplast contamination in 16S rRNA gene pyrosequencing of insect herbivore bacterial communities. J Microbiol Meth 2013; 95: 149–155. 10.1016/j.mimet.2013.08.007 PMC4133986 23968645

[43] Vero S, Garmendia G, González MB, Bentancur O, Wisniewski M. Evaluation of yeasts obtained from Antarctic soil samples as biocontrol agents for the management of postharvest diseases of apple (Malus×domestica). FEMS Yeast Res 2013; 13: 189–199. doi: 10.1111/1567-1364.12021 23136855 10.1111/1567-1364.12021

[44] Li XY, Mao ZC, Wang YH, Wu YX, He YQ, Long CL. Diversity and active mechanism of fengycin-type cyclopeptides from *Bacillus subtilis* XF-1 against *Plasmodiophora brassicae*. J Microbiol Biotechn 2013; 23: 313–321. 10.4014/jmb.1208.08065 23462003

[45] Vanneste JL, Yu J, Cornish DA, Max S, Clark G. Presence of *Pseudomonas syringae* pv. *actinidiae*, the causal agent of bacterial canker of kiwifruit, on symptomatic and asymptomatic tissues of kiwifruit. N Z Plant Protect 2011; 64: 241–245.

[46] Yeoung YR, Kim JH, Kim BS, Jeon JY, Yoon CS. Effects of beneficial antagonists (*Bacillus* sp., *Pseudomonas* sp., and *Trichoderma* sp.) on control of clubroot of Chinese cabbage. Korean J Hortic Sci 2003; 21: 194–198.

[47] Peng G, McGregor L, Lahlali R, Gossen BD, Hwang SF, Adhikari KK, et al Potential biological control of clubroot on canola and crucifer vegetable crops. Plant Pathol 2011; 60: 566–574.

[48] Cheah LH, Veerakone S, Kent G. Biological control of clubroot on cauliflower with *Trichoderma* and *Streptomyces* spp. N Z Plant Prot 2000; 53:18–21.

[49] Cheah LH, Kent G, Gowers S. Brassica crops and a *Streptomyces* sp. as potential biocontrol for clubroot of Brassicas. N Z Plant Prot 2001; 54, 80–83.

[50] Wang J, Huang Y, Lin S, Liu F, Song Q, Zhao L. A strain of *Streptomyces griseoruber* isolated from rhizospheric soil of Chinese cabbage as antagonist to *Plasmodiophora brassicae*. Ann Microbiol 2012; 62: 247–253.

[51] Hahm S, Kim J, Han K, Kim B, Kim H, Nam Y, et al Biocontrol efficacy of endophytic bacteria *Flavobacterium hercynim* EPB-C313 for control of Chinese cabbage clubroot. Research in Plant Disease 2012; 18: 210–216.

[52] Adhikari TB, Joseph CM, Yang G, Phillips DA, Nelson LM. Evaluation of bacteria isolated from rice for plant growth promotion and biological control of seedling disease of rice. Can J Microbiol 2001; 47: 916–924. 11718545 10.1139/w01-097

[53] Esitken A, Ercisli S, Karlidag H, Sahin F. Potential use of plant growth promoting rhizobacteria (PGPR) in organic apricot production. In Proceedings of the international scientific conference: Environmentally friendly fruit growing, Tartu University Press 2005.

[54] Madhaiyan M, Poonguzhali S, Senthilkumar M, Pragatheswari D, Lee JS, Lee KC. *Arachidicoccus rhizosphaerae* gen. nov., sp. nov., a plant-growth-promoting bacterium in the family Chitinophagaceae isolated from rhizosphere soil. Int J Syst Evol Micr 2015; 65: 578–586. 10.1099/ijs.0.069377-0 25404481

[55] Janisiewicz WJ, Kurtzman CP, Buyer JS. Yeasts associated with nectarines and their potential for biological control of brown rot. Yeast 2010; 27: 389–398. doi: 10.1002/yea.1763 20225339 10.1002/yea.1763

[56] Huang R, Che HJ, Zhang J, Yang L, Jiang DH, Li GQ. Evaluation of *Sporidiobolus pararoseus* strain YCXT3 as biocontrol agent of *Botrytis cinerea* on post-harvest strawberry fruits. Biol Control 2012; 62: 53–63.

[57] de Melo Pereira GV, Beux M, Pagnoncelli MG, Soccol VT, Rodrigues C, Soccol CR. Isolation, selection and evaluation of antagonistic yeasts and lactic acid bacteria against ochratoxigenic fungus *Aspergillus westerdijkiae* on coffee beans. Lett Appl Microbiol 2016; 62: 96–101. doi: 10.1111/lam.12520 26544541 10.1111/lam.12520

[58] Margesin R, Zhang DC. *Humitalea rosea* gen. nov., sp. nov., an aerobic bacteriochlorophyll-containing bacterium of the family Acetobacteraceae isolated from soil. Int J Syst Evol Micr 2013; 63: 1411–1416. 10.1099/ijs.0.043018-0 22821736

[59] Dixon GR. *Plasmodiophora brassicae* in its environment. J Plant Growth Regu 2009; 28: 212–228.

[60] Tekaia F, Latgé JP. *Aspergillus fumigatus*: saprophyte or pathogen? Curr Opin Microbiol 2005; 8: 385–392. doi: 10.1016/j.mib.2005.06.017 16019255 10.1016/j.mib.2005.06.017

