# Supplementary figures and images for: Endosphere microbiome comparison between symptomatic and asymptomatic roots of Brassica napus infected with Plasmodiophora brassicae
Source: PLoS One. 2017 Oct 24;12(10):e0185907. doi: 10.1371/journal.pone.0185907 (PMC5655474; doi:10.1371/journal.pone.0185907)

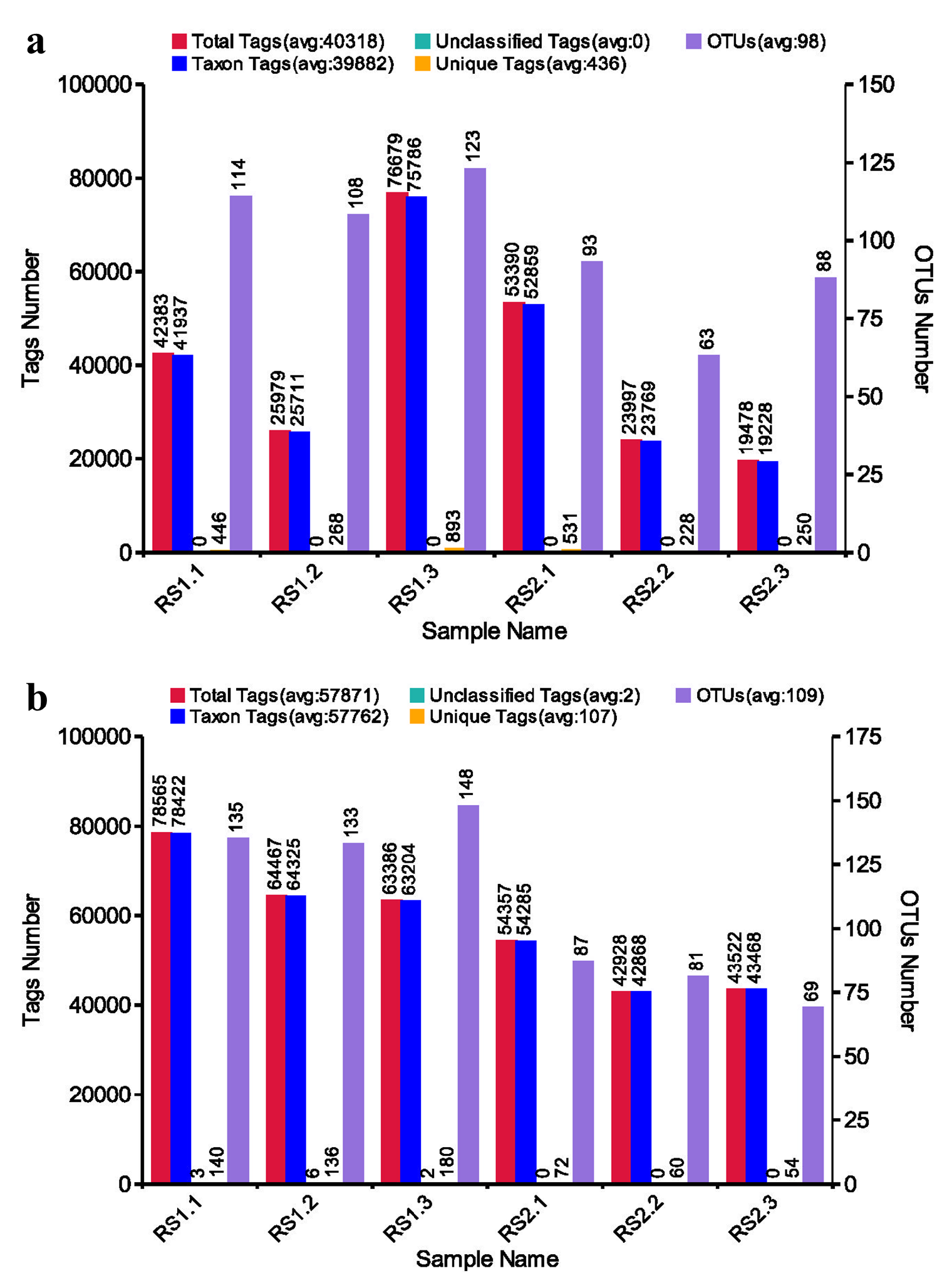

Supplement: S1 Fig — The statistical figures show the number of Tags and OTUs for all samples from the 16S rRNA sequencing results (a) and ITS sequencing results (b). Total Tags, the filtered splicing sequence numbers; Taxon Tags, the number of tags used to build the OTUs and gain classification information; Unclassified Tags, the number of tags used to build OTUs but which did not provide any classification information; Unique Tags, the number of tags for which the frequency is one and cannot be clustered into OTUs; OTUs, the final OTU numbers. (TIF) [file pone.0185907.s001.tif]

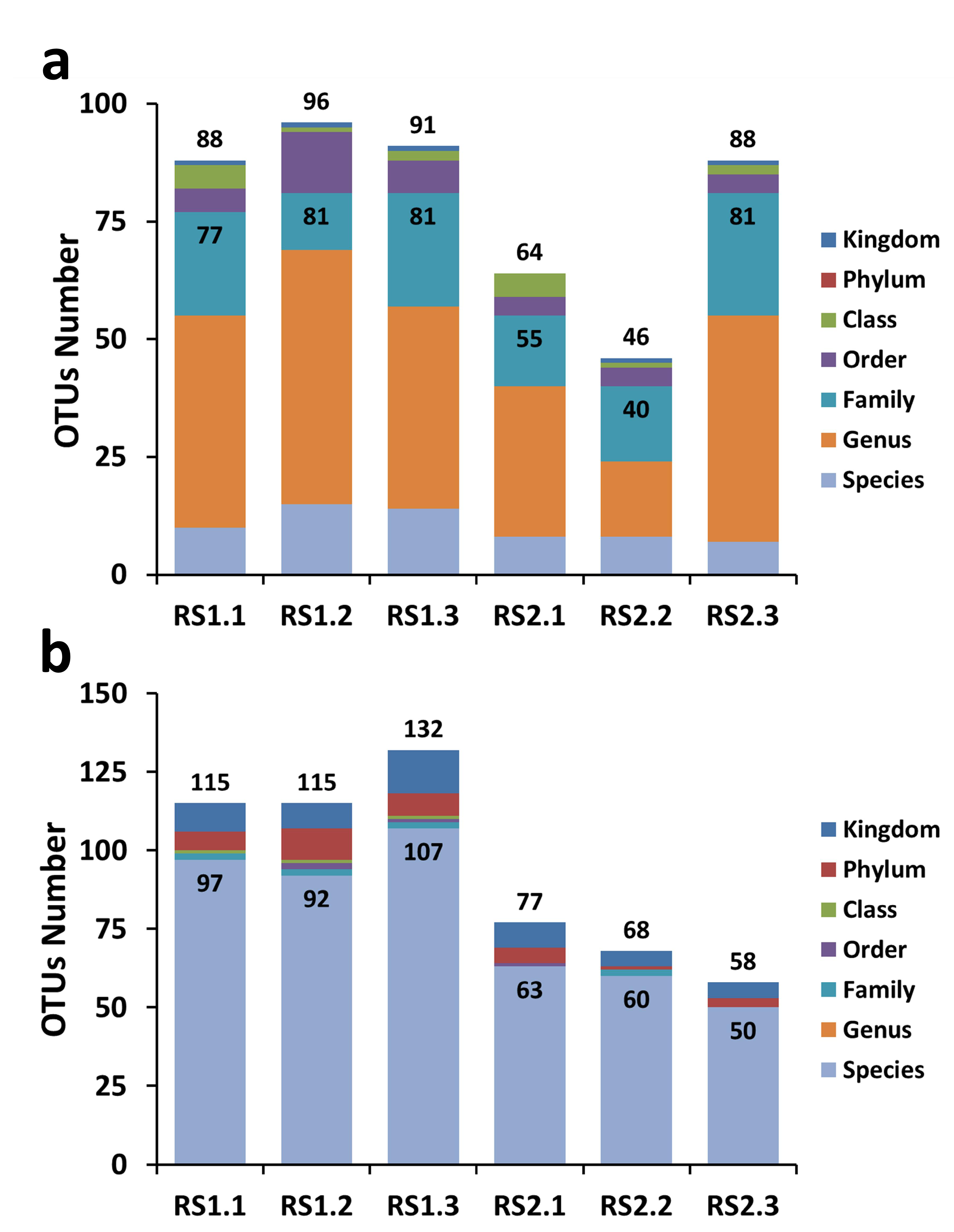

Supplement: S2 Fig — (a), The OTUs were classified by the taxonomic information into different bacterial classification levels detected in the 16S rRNA sequencing results. The above row of numbers refers to the number of OTUs classified to the bacterial Kingdom level and the lower row of numbers refers to the number of OTUs classified to the bacterial Family level. (b), The OTUs were classified by the taxonomic information into different fungal classification levels detected in the ITS sequencing results. The above row of numbers refers to the number of OTUs classified into the fungal Kingdom level and the lower row of numbers refers to the number of OTUs classified into the fungal Genus level. (TIF) [file pone.0185907.s002.tif]
