# Supplementary material for: Endosphere microbiome comparison between symptomatic and asymptomatic roots of Brassica napus infected with Plasmodiophora brassicae
Source: PLoS One. 2017 Oct 24;12(10):e0185907. doi: 10.1371/journal.pone.0185907 (PMC5655474; doi:10.1371/journal.pone.0185907)
Supplement: S1 Table — Raw data, the number of PE reads; Raw Tags, Tag number of patchwork sequence; Clean Tags: Tags taken off the low quality tag number; Effective Tags, Tag number for aftershock; Base, The number of basesof the Effective Data; AvgLen, The average length of the Effective Tags; Q30, Base percentage of the sequencing error rate is less than 0.1% in Effective Tags; Effective (%), Effective Tags/PE Reads. (DOCX) [file pone.0185907.s003.docx]

**S1 Table**

| **Sample Name** | **Raw data**  **(#)** | **Raw Tags**  **(#)** | **Clean Tags**  **(#)** | **Effective Tags**  **(#)** | **Base**  **(nt)** | **Avg**  **Len**  **(nt)** | **Q30**  **(%)** | **Effective**  **(％)** |
| --- | --- | --- | --- | --- | --- | --- | --- | --- |
| **RS1.1** | 45471 | 42675 | 42450 | **42383** | 10693537 | 252 | **97.13** | **93.21** |
| **RS1.2** | 27897 | 26146 | 26012 | **25979** | 6557127 | 252 | **97.15** | **93.12** |
| **RS1.3** | 82715 | 77217 | 76844 | **76679** | 19348424 | 252 | **96.94** | **92.7** |
| **RS2.1** | 57064 | 53742 | 53460 | **53390** | 13457980 | 252 | **97.11** | **93.56** |
| **RS2.2** | 25939 | 24158 | 24032 | **23997** | 6057323 | 252 | **96.99** | **92.51** |
| **RS2.3** | 20842 | 19618 | 19508 | **19478** | 4915752 | 252 | **97.27** | **93.46** |
