# Supplementary material for: Endosphere microbiome comparison between symptomatic and asymptomatic roots of Brassica napus infected with Plasmodiophora brassicae
Source: PLoS One. 2017 Oct 24;12(10):e0185907. doi: 10.1371/journal.pone.0185907 (PMC5655474; doi:10.1371/journal.pone.0185907)
Supplement: S2 Table — Raw data: the number of PE reads; Raw Tags: Tag number of patchwork sequence; Clean Tags: Tags taken off thelow quality tag number; Effective Tags: Tag number for aftershock; Base: The number of bases of the Effective Data; AvgLen: The average length of the Effective Tags; Q30: Base percentage of the sequencing error rate is less than 0.1%in Effective Tags; Effective (%): Effective Tags/PE Reads. (DOCX) [file pone.0185907.s004.docx]

**S2 Table**

| **Sample Name** | **Raw data**  **(#)** | **Raw Tags**  **(#)** | **Clean Tags**  **(#)** | **Effective Tags****(#)** | **Base**  **(nt)** | **Avg**  **Len**  **(nt)** | **Q30**  **(%)** | **Effective**  **(％)** |
| --- | --- | --- | --- | --- | --- | --- | --- | --- |
| **RS1.1** | 66344 | 64819 | 64691 | **64467** | 11557386 | 179 | **99.26** | **97.17** |
| **RS1.2** | 66782 | 63697 | 63582 | **63386** | 11560823 | 182 | **99.18** | **94.91** |
| **RS1.3** | 55796 | 54575 | 54477 | **54357** | 9708973 | 179 | **99.25** | **97.42** |
| **RS2.1** | 44365 | 43127 | 43041 | **42928** | 7658595 | 178 | **99.25** | **96.76** |
| **RS2.2** | 44576 | 43728 | 43640 | **43522** | 7769933 | 179 | **99.23** | **97.64** |
| **RS2.3** | 66344 | 64819 | 64691 | **64467** | 11557386 | 179 | **99.26** | **97.17** |
