# Supplementary material for: Endosphere microbiome comparison between symptomatic and asymptomatic roots of Brassica napus infected with Plasmodiophora brassicae
Source: PLoS One. 2017 Oct 24;12(10):e0185907. doi: 10.1371/journal.pone.0185907 (PMC5655474; doi:10.1371/journal.pone.0185907)
Supplement: S3 Table — (DOCX) [file pone.0185907.s005.docx]

**S3 Table.**

| Family | RS1.1 | RS1.2 | RS1.3 | RS2.1 | RS2.2 | RS2.3 |
| --- | --- | --- | --- | --- | --- | --- |
| others | 0.053072626 | 0.07028754 | 0.034852547 | 0.916401636 | 0.771929825 | 0.809578107 |
| Oxalobacteraceae | 0.268156425 | 0.257188498 | 0.193029491 | 0.029077692 | 0.060818713 | 0.070125428 |
| Pseudomonadaceae | 0.086592179 | 0.110223642 | 0.246648794 | 0.009541118 | 0.065497076 | 0.025655644 |
| Comamonadaceae | 0.117318436 | 0.121405751 | 0.134048257 | 0.011358473 | 0.021052632 | 0.015393387 |
| Xanthomonadaceae | 0.092178771 | 0.052715655 | 0.08310992 | 0.007723762 | 0.01871345 | 0.010832383 |
| Methylophilaceae | 0.036312849 | 0.067092652 | 0.021447721 | 0.007269423 | 0.009356725 | 0.031356899 |
| Rhizobiaceae | 0.053072626 | 0.04313099 | 0.034852547 | 0.003634711 | 0.004678363 | 0.00627138 |
| Flavobacteriaceae | 0.036312849 | 0.041533546 | 0.050938338 | 0.001363017 | 0.003508772 | 0.002850627 |
| Sphingomonadaceae | 0.044692737 | 0.060702875 | 0.024128686 | 0 | 0 | 0 |
| Nocardiaceae | 0.022346369 | 0.014376997 | 0.024128686 | 0.002271695 | 0.009356725 | 0.005701254 |
| Phyllobacteriaceae | 0.025139665 | 0.00798722 | 0.013404826 | 0.000908678 | 0.007017544 | 0.003990878 |
| Sphingobacteriaceae | 0.039106145 | 0.017571885 | 0.00536193 | 0.000454339 | 0 | 0.000570125 |
| Microbacteriaceae | 0.011173184 | 0.009584665 | 0.008042895 | 0.001817356 | 0.004678363 | 0.002850627 |
| Caulobacteraceae | 0.019553073 | 0.022364217 | 0.002680965 | 0.000454339 | 0.001169591 | 0.000570125 |
| Micrococcaceae | 0.016759777 | 0.006389776 | 0.021447721 | 0.000454339 | 0.002339181 | 0.000570125 |
| Enterobacteriaceae | 0 | 0.004792332 | 0.002680965 | 0.000908678 | 0.005847953 | 0.005131129 |
| Rhodocyclaceae | 0 | 0.00798722 | 0.016085791 | 0.001817356 | 0.002339181 | 0.001140251 |
| Verrucomicrobiaceae | 0.019553073 | 0.00798722 | 0.00536193 | 0 | 0 | 0 |
| Streptomycetaceae | 0.008379888 | 0.006389776 | 0.013404826 | 0 | 0 | 0 |
| Intrasporangiaceae | 0.002793296 | 0.006389776 | 0.016085791 | 0 | 0 | 0 |
| Hyphomicrobiaceae | 0.002793296 | 0.009584665 | 0.002680965 | 0.000454339 | 0 | 0.000570125 |
| Bradyrhizobiaceae | 0.005586592 | 0.006389776 | 0 | 0 | 0.003508772 | 0.000570125 |
| Opitutaceae | 0 | 0.00798722 | 0.002680965 | 0.000454339 | 0 | 0.000570125 |
| Flexibacteraceae | 0.002793296 | 0.006389776 | 0 | 0.000908678 | 0 | 0.000570125 |
| Hydrogenophilaceae | 0 | 0.003194888 | 0.00536193 | 0.001363017 | 0 | 0.000570125 |
| Nocardioidaceae | 0.005586592 | 0.009584665 | 0 | 0 | 0 | 0 |
| Pseudonocardiaceae | 0.005586592 | 0.003194888 | 0.00536193 | 0 | 0 | 0 |
| Rhodobacteraceae | 0.005586592 | 0 | 0.00536193 | 0.000454339 | 0 | 0.000570125 |
| Mycobacteriaceae | 0.005586592 | 0 | 0.008042895 | 0 | 0 | 0 |
| Peptostreptococcaceae | 0 | 0 | 0 | 0.000454339 | 0.003508772 | 0.000570125 |
| Cryomorphaceae | 0.005586592 | 0.003194888 | 0 | 0 | 0 | 0 |
| Moraxellaceae | 0 | 0 | 0 | 0 | 0.001169591 | 0.001710376 |
| auto67_4W | 0 | 0 | 0 | 0 | 0.002339181 | 0.000570125 |
| Burkholderiaceae | 0 | 0.003194888 | 0.002680965 | 0 | 0 | 0 |
| Rhodospirillaceae | 0.002793296 | 0.001597444 | 0.002680965 | 0 | 0 | 0 |
| Turicibacteraceae | 0 | 0.003194888 | 0.002680965 | 0 | 0 | 0 |
| Acetobacteraceae | 0 | 0 | 0 | 0.000454339 | 0 | 0.000570125 |
| Alteromonadaceae | 0.002793296 | 0.001597444 | 0 | 0 | 0 | 0 |
| Bacillaceae | 0 | 0.001597444 | 0.002680965 | 0 | 0 | 0 |
| Chitinophagaceae | 0.002793296 | 0 | 0.002680965 | 0 | 0 | 0 |
| Deinococcaceae | 0 | 0 | 0 | 0 | 0.001169591 | 0.000570125 |
| Nannocystaceae | 0 | 0.001597444 | 0.002680965 | 0 | 0 | 0 |
| Promicromonosporaceae | 0 | 0.001597444 | 0.002680965 | 0 | 0 | 0 |
