# Supplementary material for: Endosphere microbiome comparison between symptomatic and asymptomatic roots of Brassica napus infected with Plasmodiophora brassicae
Source: PLoS One. 2017 Oct 24;12(10):e0185907. doi: 10.1371/journal.pone.0185907 (PMC5655474; doi:10.1371/journal.pone.0185907)
Supplement: S4 Table — (DOCX) [file pone.0185907.s006.docx]

**S4 Table.**

| Genus | RS1.1 | RS1.2 | RS1.3 | RS2.1 | RS2.2 | RS2.3 |
| --- | --- | --- | --- | --- | --- | --- |
| *Olpidium* | 0.952295 | 0.974736 | 0.907693 | 0.98619 | 0.991416 | 0.989269 |
| Un-s-*Tremellomycetes* sp | 0.011127 | 0.002869 | 0.013973 | 0.001423 | 0.001096 | 0.001003 |
| others | 0.004269 | 0.002146 | 0.01325 | 0.000723 | 0.000863 | 0.000583 |
| *Tetracladium* | 0.004689 | 0.001213 | 0.003966 | 0.00161 | 0.000886 | 0.002099 |
| Un-s-*Nectriacea*e sp | 0.000863 | 0.003756 | 0.005435 | 0.00042 | 0.000303 | 7E-05 |
| Verticillium | 0.002053 | 0.0007 | 0.005225 | 0.000816 | 0.00021 | 9.33E-05 |
| *Fusarium* | 0.001983 | 0.001983 | 0.003989 | 0.000327 | 0.000257 | 0.000373 |
| *Gibberella* | 0.000257 | 0.00028 | 0.006928 | 0.001283 | 4.67E-05 | 0 |
| *Alternaria* | 0.002403 | 0.000397 | 0.004316 | 0.000327 | 0.00035 | 0.00028 |
| Un-s-*Sordariomycetes* sp | 0.003219 | 0.002146 | 0.001166 | 7E-05 | 4.67E-05 | 0.00077 |
| *Mortierella* | 0.002309 | 0.00084 | 0.00154 | 0.000513 | 0.000886 | 0.000537 |
| Un-s-Fungi sp | 0.001516 | 0.00063 | 0.002893 | 0.00028 | 0.000163 | 9.33E-05 |
| Un-s-fungal sp WEF9 | 4.67E-05 | 4.67E-05 | 0.004549 | 0 | 0 | 0 |
| Un-s-leaf litter ascomycete strain its348 | 0.000163 | 0.000233 | 0.003732 | 0 | 0 | 0 |
| *Blastobotrys* | 0.002613 | 0.000117 | 4.67E-05 | 4.67E-05 | 2.33E-05 | 0.00028 |
| Un-s-fungal endophyte sp AP053 | 0.000257 | 0.00021 | 0.001936 | 0.00035 | 0.000163 | 9.33E-05 |
| *Tetraplosphaeria* | 2.33E-05 | 0 | 0.002869 | 0 | 0 | 0 |
| *Acremonium* | 0.000117 | 0.000163 | 0.00161 | 4.67E-05 | 0.00028 | 0.000117 |
| *Rhodotorula* | 0.000117 | 0.001773 | 2.33E-05 | 0.000117 | 4.67E-05 | 0.000117 |
| *Sclerotinia* | 0.000653 | 7E-05 | 0.000863 | 0.000117 | 0.000303 | 0.000187 |
| *Articulospora* | 0.000746 | 0.000233 | 9.33E-05 | 9.33E-05 | 4.67E-05 | 0.000886 |
| *Podospora* | 0.00035 | 4.67E-05 | 0.00098 | 0.00014 | 0.000257 | 0.000233 |
| *Boeremia* | 0.000886 | 0.000327 | 0.000187 | 2.33E-05 | 4.67E-05 | 0.00035 |
| *Plectosphaerella* | 0.00049 | 0.000257 | 0.00084 | 4.67E-05 | 4.67E-05 | 0.000117 |
| Un-s-cf *Acremonium* sp SS_1583 | 0.000233 | 4.67E-05 | 0.001516 | 0 | 0 | 0 |
| *Phaeoseptoria* | 0.000117 | 2.33E-05 | 0.001236 | 0 | 0 | 0 |
| *Cistella* | 2.33E-05 | 0.00014 | 0.001073 | 0 | 0 | 0 |
| Un-s-*Hypocreales* sp | 0.000886 | 4.67E-05 | 0.000233 | 0 | 0 | 0 |
| Un-s-fungal endophyte | 0.000583 | 0.000187 | 0.00014 | 7E-05 | 2.33E-05 | 0.000117 |
| Khuskia | 7E-05 | 2.33E-05 | 0.000863 | 9.33E-05 | 2.33E-05 | 0 |
| Un-s-*Mortierellales* sp | 0.00021 | 0.000117 | 0.000373 | 0.000163 | 4.67E-05 | 0 |
| Un-s-*Dothideomycetes* sp | 0.000163 | 0.00014 | 0.000163 | 0.00021 | 0.00014 | 7E-05 |
| Un-s-*Sporidiobolales* sp | 0.00014 | 0.00014 | 0.000257 | 0.000117 | 0 | 2.33E-05 |
| *Scleroderma* | 4.67E-05 | 0.000117 | 0.00021 | 2.33E-05 | 0.000187 | 4.67E-05 |
| Un-s-fungal sp 3 EO_2010 | 0.000163 | 0.00014 | 0.000163 | 0 | 9.33E-05 | 7E-05 |
| Un-s-*Ascomycota* sp | 0.000163 | 0.000163 | 7E-05 | 4.67E-05 | 4.67E-05 | 9.33E-05 |
| Un-s-*Tremellales* sp | 0.000327 | 9.33E-05 | 4.67E-05 | 7E-05 | 4.67E-05 | 0 |
| Un-s-*Auriculariales* sp | 4.67E-05 | 4.67E-05 | 0 | 0.000257 | 0.00021 | 0 |
| Un-s-*Phaeosphaeriaceae* sp | 4.67E-05 | 0 | 4.67E-05 | 0.000443 | 0 | 2.33E-05 |
| *Phoma* | 0.000327 | 0.00014 | 2.33E-05 | 0 | 0 | 0 |
| Un-s-*Pleosporales* sp | 0.000233 | 0.000163 | 2.33E-05 | 0 | 0 | 0 |
| *Sporobolomyces* | 0.00014 | 2.33E-05 | 0.000187 | 2.33E-05 | 0 | 4.67E-05 |
| *Ilyonectria* | 0.00014 | 0.000163 | 9.33E-05 | 0 | 0 | 0 |
| *Lulworthia* | 0.000117 | 7E-05 | 2.33E-05 | 7E-05 | 9.33E-05 | 2.33E-05 |
| *Perisporiopsis* | 9.33E-05 | 0 | 0.000257 | 0 | 0 | 0 |
| Un-s-*Agaricales* sp | 0.000233 | 4.67E-05 | 2.33E-05 | 0 | 0 | 0 |
| Un-s-*Helotiales* sp | 9.33E-05 | 0.000163 | 4.67E-05 | 0 | 0 | 0 |
| *Leptosphaeria* | 0.000163 | 0.000117 | 0 | 0 | 0 | 0 |
| *Paecilomyces* | 0.000163 | 0 | 0.000117 | 0 | 0 | 0 |
| *Sporidiobolus* | 0.00014 | 0 | 0.000117 | 0 | 0 | 0 |
| *Myrmecridium* | 2.33E-05 | 0 | 0.00021 | 0 | 0 | 0 |
| *Glomus* | 2.33E-05 | 7E-05 | 0.000117 | 0 | 0 | 0 |
| *Leucosporidium* | 0.00014 | 2.33E-05 | 4.67E-05 | 0 | 0 | 0 |
| *Myrothecium* | 2.33E-05 | 2.33E-05 | 0 | 7E-05 | 0 | 9.33E-05 |
| *Ceratocystis* | 7E-05 | 2.33E-05 | 9.33E-05 | 0 | 0 | 0 |
| Un-s-*Pleosporales* sp REF107 | 0 | 0 | 0 | 0 | 0.00014 | 4.67E-05 |
| *Mastigobasidium* | 4.67E-05 | 0.000117 | 0 | 0 | 0 | 0 |
| *Aspergillus* | 0 | 0 | 0 | 0 | 4.67E-05 | 9.33E-05 |
| *Cylindrocarpon* | 0 | 7E-05 | 4.67E-05 | 0 | 0 | 0 |
| *Cylindrotrichum* | 0 | 7E-05 | 4.67E-05 | 0 | 0 | 0 |
| Un-s-*Chaetomiaceae* sp | 2.33E-05 | 0 | 4.67E-05 | 0 | 2.33E-05 | 2.33E-05 |
| Un-s*-Mortierellaceae* sp | 2.33E-05 | 0 | 4.67E-05 | 0 | 2.33E-05 | 2.33E-05 |
| Un-s-*Sebacinaceae* sp | 0 | 4.67E-05 | 7E-05 | 0 | 0 | 0 |
| *Ambispora* | 7E-05 | 2.33E-05 | 0 | 0 | 0 | 0 |
| *Clonostachys* | 2.33E-05 | 0 | 7E-05 | 0 | 0 | 0 |
| *Cryptococcus* | 7E-05 | 0 | 2.33E-05 | 0 | 0 | 0 |
| Un-s-fungal sp DG16 | 0 | 0 | 0 | 7E-05 | 2.33E-05 | 0 |
| *Bionectria* | 0 | 4.67E-05 | 2.33E-05 | 0 | 0 | 0 |
| Un-s-*Helotiales* sp r427 | 0 | 0 | 0 | 2.33E-05 | 4.67E-05 | 0 |
| Un-s-*Sordariales* sp | 4.67E-05 | 2.33E-05 | 0 | 0 | 0 | 0 |
| Un-s-*Clavulinaceae* sp | 2.33E-05 | 0 | 2.33E-05 | 0 | 0 | 0 |
